# Pyogenic liver abscess in a diabetic patient with fever of unknown origin

**DOI:** 10.1186/1471-2334-13-S1-P97

**Published:** 2013-12-16

**Authors:** Adina Ilie, Ramona Popescu, Elisabeta Stoica

**Affiliations:** 1 National Institute for Infectious Diseases “Prof. Dr. Matei Balş”, Bucharest, Romania

## Background

We present the management of febrile syndrome in a diabetic patient.

## Case report

The patient was admitted to our ward from a county hospital to establish the diagnosis of a 25-day fever and cough. The following diagnoses had been excluded: HIV, endocarditis, pneumonia, urinary tract infection and the infection appeared not to respond to ampicillin- sulbactam or levofloxacin. However, an abdominal ultrasound had not been performed.

From the medical history we noted that the patient had poorly managed diabetes mellitus type 2 and hypertension with mild cardiac disease.

We performed an abdominal ultrasound and identified a large liver abscess, confirmed by contrast enhanced CT scanning.

The patient was treated with antibiotics and after 14 days the abscess was drained under CT guidance. The etiology was established to be *E coli*.

At the present the patient is doing very well – he is still under antibiotic treatment with drain tube and ultrasound monitoring.

## Conclusion

The diagnosis in a patient with fever of unknown origin and cough turned out to be pyogenic liver abscess with good evolution under antibiotic treatment and CT guided drainage.

